# Supplementary material for: Unnatural amino acid photo-crosslinking of the IKs channel complex demonstrates a KCNE1:KCNQ1 stoichiometry of up to 4:4
Source: eLife. 2016 Jan 23;5:e11815. doi: 10.7554/eLife.11815 (PMC4807126; doi:10.7554/eLife.11815)
Supplement: Figure 1—source data 1. — DOI: http://dx.doi.org/10.7554/eLife.11815.004 [file elife-11815-fig1-data1.docx]

Figure 1 – Source data 1

V_1/2_ of activation for *I_Ks_* channel complexes

| *I_Ks_* construct | V_1/2_  (mV) | k-factor | n  (cells) |
| --- | --- | --- | --- |
| KCNQ1 | -15.5 ± 2.1 | 15.9 ± 1.1 | 5 |
| KCNQ1 + KCNE1-GFP | 26.1 ± 2.2 | 23.1 ± 1.4 | 11 |
| EQQQQ | -0.9 ± 2.0 | 19.8 ± 1.2 | 5 |
| EQQQQ + KCNE1-GFP | 30.9 ± 1.4 | 18.0 ± 1.6 | 5 |
| EQQ | 11.6 ± 0.8 | 20.0 ± 2.8 | 3 |
| EQQ + KCNE1-GFP | 31.9 ± 3.0 | 20.2 ± 1.4 | 4 |
| EQ | 31.5 ± 0.5 | 20.0 ± 1.2 | 3 |
| EQ + KCNE1-GFP | 33.4 ± 1.7 | 17.0 ± 1.0 | 5 |
