# Supplementary material for: Unnatural amino acid photo-crosslinking of the IKs channel complex demonstrates a KCNE1:KCNQ1 stoichiometry of up to 4:4
Source: eLife. 2016 Jan 23;5:e11815. doi: 10.7554/eLife.11815 (PMC4807126; doi:10.7554/eLife.11815)
Supplement: Figure 4—source data 1. — DOI: http://dx.doi.org/10.7554/eLife.11815.011 [file elife-11815-fig4-data1.docx]

Figure 4 - figure supplement 2 - Source data 1.

V_1/2_ of activation for *I_Ks_* channel complexes using 31 aa (sub S) or 52 aa (sub L) linker

| *I_Ks_* construct | V_1/2_  (mV) | k-factor | n  (cells) |
| --- | --- | --- | --- |
| KCNQ1 | -15.5 ± 2.1 | 15.9 ± 1.1 | 5 |
| KCNQ1 + KCNE1-GFP | 26.1 ± 2.2 | 23.1 ± 1.4 | 11 |
| KCNQ1 + F57Bpa KCNE1-GFP | 28.2 ± 6.8 | 25.9 ± 3.5 | 3 |
| EQQQQ_L_ | -0.9 ± 2.0 | 19.8 ± 1.2 | 5 |
| EQQQQ_L_ + KCNE1-GFP | 30.9 ± 1.4 | 18.0 ± 1.6 | 5 |
| EQQQQ_L_ + F57Bpa KCNE1-GFP | 27.7 ± 1.5 | 20.7 ± 1.7 | 3 |
| EQQ_S_ | 12.6 ± 2.7 | 19.4 ± 0.8 | 6 |
| EQQ_S_ + KCNE1-GFP | 27.9 ± 1.4 | 22.2 ± 0.9 | 9 |
| EQQ_S_ + F57Bpa KCNE1-GFP | 28.3 ± 5.4 | 23.4 ± 1.3 | 7 |
| EQ_S_ | 27.9 ± 3.1 | 23.2 ± 0.9 | 16 |
| EQ_S_ + KCNE1-GFP | 28.1 ± 2.8 | 21.6 ± 1.3 | 8 |
| EQ_S_ + F57Bpa KCNE1-GFP | 26.1 ± 1.7 | 17.3 ± 1.2 | 3 |
