# Supplementary material for: Unnatural amino acid photo-crosslinking of the IKs channel complex demonstrates a KCNE1:KCNQ1 stoichiometry of up to 4:4
Source: eLife. 2016 Jan 23;5:e11815. doi: 10.7554/eLife.11815 (PMC4807126; doi:10.7554/eLife.11815)
Supplement: Figure 5—source data 1. — DOI: http://dx.doi.org/10.7554/eLife.11815.015 [file elife-11815-fig5-data1.docx]

Figure 5 – Source data 1

Crosslinking rate constants for *I_Ks_* channel complexes

| *I_Ks_* construct | K_XL_  (s^-1^) | n  (cells) |
| --- | --- | --- |
| KCNQ1 + F57Bpa KCNE1-GFP | 1.12 ± 0.09 | 6 |
| EQQQQ + F57Bpa KCNE1-GFP | 0.63 ± 0.06 | 6 |
| EQQ + F57Bpa KCNE1-GFP | 0.55 ± 0.06 | 7 |
| EQ + F57Bpa KCNE1-GFP | 0.09 ± 0.09 | 5 |
