# Supplementary material for: Unnatural amino acid photo-crosslinking of the IKs channel complex demonstrates a KCNE1:KCNQ1 stoichiometry of up to 4:4
Source: eLife. 2016 Jan 23;5:e11815. doi: 10.7554/eLife.11815 (PMC4807126; doi:10.7554/eLife.11815)
Supplement: Figure 5—source data 2. — DOI: http://dx.doi.org/10.7554/eLife.11815.016 [file elife-11815-fig5-data2.docx]

Figure 5 – Source data 2

Rate constants for *I_Ks_* channel complex rundown

| *I_Ks_* construct | K_RD_  (s^-1^) | n  (cells) |
| --- | --- | --- |
| KCNQ1 + KCNE1-GFP | 0.1864 ± 0.03 | 5 |
| EQQQQ + KCNE1 | 0.1588 ± 0.04 | 3 |
| EQQ + KCNE1-GFP | 0.1049 ± 0.01 | 3 |
| EQQ | 0.1849 ± 0.05 | 4 |
| EQ + KCNE1-GFP | 0.1259 ± 0.03 | 4 |
| EQ | 0.1021 ± 0.02 | 4 |
